# Evaluation of airway management associated hands-off time during cardiopulmonary resuscitation: a randomised manikin follow-up study

**DOI:** 10.1186/1757-7241-21-10

**Published:** 2013-02-25

**Authors:** Christina Gruber, Sabine Nabecker, Philipp Wohlfarth, Anita Ruetzler, Dominik Roth, Oliver Kimberger, Henrik Fischer, Michael Frass, Kurt Ruetzler

**Affiliations:** 1 Department of General Anaesthesia and Intensive Care, Medical University of Vienna, Vienna, Austria; 2 Department of Cardiothoracic and Vascular Anaesthesia and Intensive Care Medicine, Medical University of Vienna, Vienna, Austria; 3 Social Medical Center East, Vienna, Austria; 4 Department of Emergency Medicine, Medical University of Vienna, Vienna, Austria; 5 Department of Medicine I, Medical University of Vienna, Vienna, Austria; 6 Institute of Anaesthesiology, University Hospital Zuerich, Raemistrasse 100, Zuerich 8091, Switzerland

**Keywords:** Anaesthesia, Emergency medical technicians, Hands-off time, Endotracheal intubation, Supraglottic airways, Emergency airway management, CPR

## Abstract

**Introduction:**

Airway management is an important component of cardiopulmonary resuscitation (CPR). Recent guidelines recommend keeping any interruptions of chest compressions as short as possible and not lasting more than 10 seconds. Endotracheal intubation seems to be the ideal method for establishing a secure airway by experienced providers, but emergency medical technicians (EMT) often lack training and practice. For the EMTs supraglottic devices might serve as alternatives.

**Methods:**

40 EMTs were trained in a 1-hour standardised audio-visual lesson to handle six different airway devices including endotracheal intubation, Combitube, EasyTube, I-Gel, Laryngeal Mask Airway and Laryngeal tube. EMTs performances were evaluated immediately after a brief practical demonstration, as well as after 1 and 3 months without any practice in between, in a randomised order. Hands-off time was pair-wise compared between airway devices using a repeated-measures mixed-effects model.

**Results:**

Overall mean hands-off time was significantly (p<0.01) lower for Laryngeal tube (6.1s; confidence interval 5.2-6.9s), Combitube (7.9s; 95% CI 6.9-9.0s), EasyTube (8.8s; CI 7.3-10.3s), LMA (10.2s; CI 8.6-11.7s), and I-Gel (11.9s; CI 10.2-13.7s) compared to endotracheal intubation (39.4s; CI 34.0-44.9s). Hands-off time was within the recommended limit of 10s for Combitube, EasyTube and Laryngeal tube after 1 month and for all supraglottic devices after 3 months without any training, but far beyond recommended limits in all three evaluations for endotracheal intubation.

**Conclusion:**

Using supraglottic airway devices, EMTs achieved a hands-off time within the recommended time limit of 10s, even after three months without any training or practice. Supraglottic airway devices are recommended tools for EMTs with lack of experience in advanced airway management.

## Introduction

Any delay in initiating chest compressions during cardiopulmonary resuscitation (CPR) reduces coronary and cerebral perfusion. Therefore, early chest compressions and establishment of a secure airway as soon as possible are recommended by the European Resuscitation Council (ERC) and the American Heart Association (AHA) [1,2]. Interruptions of chest compressions during CPR, represented by the so called “hands-off” time, should be kept as short as possible [2,3]. ERC guidelines suggest that skilled clinicians should be able to secure the airway fully without interrupting chest compressions or within a brief pause not exceeding 10 seconds [2]. A minimal hands-off time was shown to result in an up to 3-fold increase of survival of out of hospital cardiopulmonary arrest [4].

In experienced hands, endotracheal intubation seems to be the optimal method for providing and maintaining a clear and secure airway during CPR [3]. Indeed, endotracheal intubation requires a high level of experience and regular re-training [5,6]. If conventional endotracheal intubation is performed rarely, up to 50% of intubation attempts will fail or will need repeated efforts [7]. Furthermore, repeated and prolonged laryngoscopy attempts are well-known contributors to morbidity and mortality [8]. Due to these facts and other research data, performing endotracheal intubation in the pre-hospital emergency setting has been questioned recently [9-11].

Results of previous studies showed that supraglottic airway devices are easier to insert compared to endotracheal intubation, even during on-going CPR and in unskilled hands [12-14]. Therefore, ERC guidelines recommend supraglottic devices such as the Laryngeal Mask Airway (LMA) and the Combitube as alternatives to endotracheal intubation, especially in inexperienced hands [2].

In many countries in Europe, practical experience of Emergency Medical Technicians (EMT) with conventional endotracheal intubation is low [15]. Therefore, we hypothesised that hands-off time for airway management during CPR would be significantly lower using supraglottic airway devices compared to conventional endotracheal intubation, when performed by inexperienced EMT`s and after a period without training.

## Methods

Following approval by the local Ethics Committee of the Medical University of Vienna, we recruited 40 active voluntary EMT`s of the Red Cross, regional association Burgenland, Austria without any advanced airway management training. In Austria, EMT`s are only educated in basic airway management skills including mouth-to-mouth and bag-valve ventilation. For advanced airway management, such as endotracheal intubation or placing a supraglottic airway device, further advanced education is required. Results of initial evaluation have already been published [12].

After having given written consent, all EMT`s participated in an 1-hour standardised audio-visual lecture. The topics covered relevant aspects of anatomy, CPR and different techniques for securing the airway. The lecture was followed by a practical demonstration of insertion, cuff inflation and subsequent ventilation with a bag using each of the following devices:

1. Laryngoscope guided endotracheal tube 7.5 mm I.D. (Mallinckrodt, Athlone, Ireland), reinforced with a stylet;

2. Combitube SA 37 F (Covidien, Mansfield, MA, USA);

3. EasyTube Ch 41 (Teleflex Medical Ruesch, Research Triangle Park, NC, USA);

4. I-Gel size 4 (Intersurgical Ltd., Wokingham, England);

5. Laryngeal Tube disposable size 4 (King LT-D, VBM, Sulz, Germany);

6. Laryngeal Mask unique – LMA size 4 (LMA Company North America, San Diego, CA, USA);

For practical demonstration the Resusci Anne Advanced Simulator^®^ (Laerdal Medical, Stavanger, Norway) was used.

After practical demonstration the performances of all EMT`s were analysed in a separate room. Two EMT`s formed a CPR team. One EMT performed chest compressions, while the other member was responsible for airway management. Each team performed basic CPR in the usual manner (ratio 30:2), using bag-valve ventilation for two minutes. Afterwards, the EMT responsible for airway management used the six airway devices listed above in a randomised order. The random sample was generated by the ARandomizer Software (https://www.meduniwien.ac.at/randomizer/web/login.php.). The manikin and the airway devices were lubricated with lubricant recommended by Laerdal Medical.

The EMT`s were strictly advised to minimise interruptions of chest compression for airway management. If possible, airway management was performed during on-going chest compressions. If necessary, interruption of chest compressions was kept as short as possible, but not exceeding 10 seconds. For attempts lasting longer than 10 seconds, the EMT`s were instructed to stop airway-management and start instead with bag-valve ventilation in the ratio 30:2 for 2 minutes. The number of insertion attempts for each device was limited to three attempts.

Practical performance was finished after announcement of successful insertion by the EMT or after three intubation attempts. Unsuccessful airway management was defined either as unrecognised oesophageal intubation or, if participants were unable to insert the device within 3 attempts. The EMT`s were allowed to correct the position, if misplacement of the airway device was recognised (by absent or inadequate chest movements).

One and three months later, all EMT`s participated in a second and a third evaluation using the identical study setting and equipment without any further theoretical or practical training.

The primary outcome was to determine the average hands-off time over all three evaluations. Hands-off time was defined as the cumulative duration of CPR discontinuation during airway insertion. Interruptions lasting longer than 1.5 seconds were considered as the beginning of hands-off time. Hands-off time was automatically recorded by the manikin`s computer.

The secondary outcomes were to determine the average number of intubation attempts, overall success rate (defined as correct placement of the device within a maximum of three attempts), and to record unrecognised misplacement. Data of secondary outcomes were recorded by the investigators.

### Statistical analysis

A single CPR session (i.e. single evaluation of one EMT using one device) was used as the unit of analysis. We calculated repeated-measures mixed-effects models for pair-wise comparison between airway device groups (endotracheal intubation compared with each of the other devices) and paired t-test for comparison between different evaluations (first evaluation vs. second and third evaluation) of hands-off time and number of attempts, respectively. Furthermore, we used the chi-square test for analysing the percentage of successful intubations. The correction for multiple testing for all tests was performed according to Sidak [16]. Results are reported as the mean average (95% confidence intervals (CI)). A p-value of less than 0.05 was considered statistically significant. Overall success rate and unrecognised misplacements are reported as the proportion of total number of CPR sessions for each device.

Based on the results of a previous study, we expected an average difference in hands-off time of 30% with a standard deviation of ± 30% between endotracheal intubation and supraglottic airway devices [12]. To show this difference with an alpha error of 0.05 and a power of 90% we needed to have a minimum of at least 30 participants. In order to take into account any potential loss of participants we included 40 EMT`s in this study. Gpower 3.1.4 (University of Kiel, Germany) was used for power calculation. Stata IC 10 (Stata Corp., College Station, Texas, USA) was used for all other analyses.

## Results

This study was conducted between January and April 2011. Forty EMT`s (33 men and 7 women, age 26 ± 5 years) participated in all three evaluations, resulting in a total number of 120 CPR sessions for each device or 720 CPR sessions in total. Three CPR sessions (at first evaluation one Combitube, and at second evaluation one Combitube and one EasyTube) had to be excluded, because of technical problems with the cuffs (destroyed cuffs during previous intubation), resulting in a net of 717 CPR sessions available for analysis.

The median of overall hands-off time was significantly higher for endotracheal intubation (39.4s; 95% CI 34.0-44.9s) compared to Laryngeal tube (6.1s; 5.2-6.9s), Combitube (7.9s; 6.9-9.0s), LMA (10.2s; 8.6-11.7s), EasyTube (8.8s; 7.3-10.3s), and I-Gel (11.9s; 10.2-13.7s) (Table 1).

**Table 1:** Skill performance at first, second and third evaluation

	**First evaluation**	**Second evaluation**	**Third evaluation**	**Overall**
**Endotracheal intubation**				
Hands-off time	48.0 (43.0-53.0)	32.9 (25.3-40.6)**	37.4 (27.7-47.1)*	39.4 (34.0-44.9)
Attempts	2.2 (1.9-2.5)	1.8 (1.5-2.0)	1.8 (1.5-2.1)	1.9 (1.8-2.1)
Successful	14/40 (35%)	23/40 (58%)	28/40 (70%)	65/120 (54%)
Unrecognised misplacement	8/40 (20%)	7/40 (18%)	6/40 (15%)	21/120 (18%)
Successful attempts within 10s	1/40 (2.5%)	6/40 (15%)*	6/40 (15%)*	13/120 (11%)
**Combitube**				
Hands-off time	10.0 (4.9-15.1)	8.2 (6.5-9.9)	6.0 (4.3-7.8)**	7.9 (6.9-9.0)
Attempts	1.3 (1.1-1.5)	1.2 (1.1-1.4)	1.0 (1.0-1.0)	1.1 (1.1-1.2)
Successful	39/39 (100%)	39/39 (100%)	40/40 (100%)	118/118 (100%)
Unrecognised misplacement	0/39 (0%)	0/39 (0%)	0/40 (0%)	0/118 (0%)
Successful attempts within 10s	29/39 (74.4%)	30/39 (76.9%)	37/40 (92.5%)*	96/118 (81%)
**EasyTube**				
Hands-off time	11.4 (6.4-16.4)	8.3 (5.3-11.4)	6.7 (4.8-8.6)**	8.8 (7.3-10.3)
Attempts	1.3 (1.1-1.5)	1.2 (1.1-1.4)	1.1 (1.0-1.1)	1.2 (1.1-1.3)
Successful	40/40 (100%)	39/39 (100%)	40/40 (100%)	119/119 (100%)
Unrecognised misplacement	0/40 (0%)	0/39 (0%)	0/40 (0%)	0/119 (0%)
Successful attempts within 10s	27/40 (67.5%)	33/39 (84.6%)	34/40 (85%)	94/119 (79%)
**I-Gel**				
Hands-off time	15.9 (10.8-20.9)	12.0 (9.6-14.4)	7.9 (6.2-9.6)**	11.9 (10.2-13.7)
Attempts	1.9 (1.6-2.2)	1.6 (1.4-1.9)	1.3 (1.1-1.5)	1.6 (1.5-1.8)
Successful	40/40 (100%)	39/40 (98%)	40/40 (100%)	119/120 (99%)
Unrecognised misplacement	0/40 (0%)	0/40 (0%)	0/40 (0%)	0/120 (0%)
Successful attempts within 10s	16/40 (40%)	18/40 (45%)	27/40 (67.5%)**	61/120 (51%)
**LMA**				
Hands-off time	13.3 (8.2-18.3)	10.3 (7.7-13.0)	6.8 (5.4-8.3)**	10.2 (8.6-11.7)
Attempts	1.6 (1.3-1.8)	1.4 (1.2-1.6)	1.3 (1.1-1.4)	1.4 (1.3-1.5)
Successful	40/40 (100%)	40/40 (100%)	40/40 (100%)	120/120 (100%)
Unrecognised misplacement	0/40 (0%)	1/40 (3%)	0/40 (0%)	1/120 (1%)
Successful attempts within 10s	21/40 (52.5%)	24/40 (60%)	33/40 (82.5%)**	78/120 (65%)
**Laryngeal tube**				
Hands-off time	8.4 (3.4-16.4)	5.2 (4.2-6.3)**	4.5 (3.7-5.3)**	6.1 (5.2-6.9)
Attempts	1.4 (1.2-1.6)	1.2 (1.0-1.3)	1.1 (1.0-1.1)	1.2 (1.1-1.3)
Successful	40/40 (100%)	40/40 (100%)	40/40 (100%)	120/120 (100%)
Unrecognised misplacement	0/40 (0%)	0/40 (0%)	0/40 (0%)	0/120 (0%)
Successful attempts within 10s	31/40 (77.5%)	39/40 (97.5%)**	39/40 (97.5%)**	109/112 (97%)

Times are presented in seconds as averages (95% confidence interval). Attempts are presented as absolute numbers (95% confidence interval). Successful airway management and misplacements are presented as proportions of CPR sessions (percentages).

*: p< 0.05 hands off time compared with the first evaluation (paired t-test).

**:p<0.025 (corrected to Sidak) hands-off time compared with the first evaluation (paired t-test).

**Figure 1:**
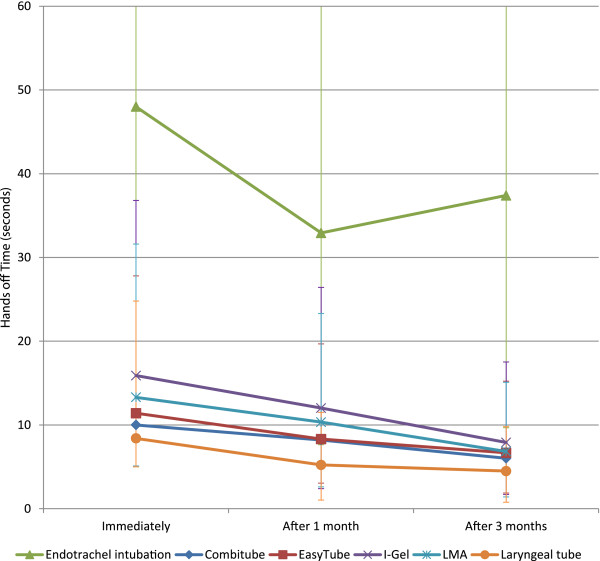
**Hands-off time.** Hands off time of different airway devices at different timepoints. Dots denote mean. LMA: Laryngeal Mask Airway.

Median hands-off time was significantly shorter in the second and third evaluation compared to initial evaluation using conventional endotracheal intubation (48.0s vs. 32.9s vs. 37.4s) and Laryngeal tube (8.4s vs. 5.2s vs. 4.5s). Other supraglottic airway devices showed a significant difference of median hands-off time comparing the first and the third evaluation. In the second evaluation there was no significant difference to initial phase (Figure 1).

The percentage of successful attempts for airway management within 10 seconds significantly increased for conventional endotracheal intubation (2.5% vs. 15% vs. 15%), the Laryngeal tube (77.5% vs. 97.5% vs. 97.5%), Combitube (74.4% vs. 76.9% vs. 92.5%), LMA (52.5% vs. 60% vs. 82.5%) and I-Gel (40% vs. 45% vs. 67.5%). EasyTube was 100% successfully inserted within 10s (Table 1).

Mean number of attempts for conventional endotracheal intubation was 1.9 (95% CI 1.8-2.1), which was significantly higher compared to Combitube (1.1; 1.1-1.2), EasyTube (1.2; 1.1-1.3), Laryngeal tube (1.2; 1.1-1.3), LMA (1.4; 1.3-1.5), and I-Gel (1.6; 1.5-1.8).

During first evaluation, conventional endotracheal intubation was correctly performed 14 times, representing a success rate of 35%. Success rate increased to 23 (58%) and 28 successful insertions (70%) during second and third evaluation. Unrecognised misplacements occurred in 8 (20%), 7 (18%) and 6 (15%) intubation attempts. All supraglottic airway devices were successfully inserted in all three evaluations by all EMT`s, with the exception of one LMA unrecognised misplacement, and one recognised unsuccessful I-Gel insertion during second evaluation (Table 1).

## Discussion

To our knowledge, our study is the first evaluating airway management associated hands-off time during CPR by EMT`s observing current CPR guidelines. Our main finding is that EMT`s successfully performed airway management using supraglottic airway devices within acceptable hands-off time, whereas conventional endotracheal intubation failed - also after several months without any theoretical or practical training.

The hands-off time required for endotracheal intubation far exceeded current CPR guidelines. Although average hands-off time decreased during the study period, median hands-off time was still significantly beyond the recommended time frame. Only 15% of intubation attempts were within the time frame during the third evaluation. Success rate of conventional endotracheal intubation increased up to 70% during the third evaluation, but 15% of all intubation attempts led to unrecognised misplacement.

Intubation in the pre-hospital setting is challenging. Therefore, extensive training is required. However, a recent study by Frascone et al. reported a maximum success rate of any 80% by using conventional endotracheal intubation, even in the hands of experienced paramedics [17]. Nicholl et al. reported a failure rate of 65% by experienced paramedics [18]. Furthermore, Sayre et al. described unsuccessful intubation at a rate of 50% and unrecognised oesophageal intubations in 3%, covering 103 pre-hospital conventional endotracheal intubations by EMT`s [19].

All supraglottic airway devices – Laryngeal Mask Airway, I-Gel, Laryngeal Tube, Combitube and EasyTube - performed well and were successfully inserted by the EMT`s. Although up to three intubation attempts were necessary, all supraglottic airway devices were successfully inserted, and the average number of intubation attempts was significantly lower, compared to conventional endotracheal intubation. Median hands-off time of supraglottic devices ranged from 6.1 to 11.9 seconds. Edelson et al. reported that a 5-second decrease in pre-shock pause is associated with an 86% increase in the odds of shock success [20].

The results of our study are consistent with the finding of Schalk et al. and Länkimäki et al. The authors reported a success rate up to 98% for pre-hospital intubations, using a Laryngeal tube [21,22]. However, the authors reported insertion times, instead of hands-off time. Wiese et al. also found comparable results in a manikin study, involving 50 intensive care nurses [23]. The Laryngeal tube could be inserted quickly and reliably during CPR.

Airway management during CPR with the LMA is successful, even with minimal experience in airway management [24,25]. In our study, only 1 out of 120 intubation attempts with LMA failed. We therefore agree that the LMA is an essential alternative airway device, even during on-going chest compressions.

The LMA and the I-Gel tended to rotate and displace laterally during CPR as previously described by Gatward et al [26]. These problems were observed by the researchers, based on researchers clinical experience and acoustic leakage noises. However, airway management was successful allowing three intubation attempts within acceptable short hands-off time in all cases. The high success rate was already demonstrated by Wharton et al. and Wiese et al. [27,28] Recently, Theiler et al. reported an overall success rate of 96% covering 2049 I-gel insertions in clinical practice [29]. Although not investigated during CPR by Theiler et al. the results support the findings, that the I-Gel might also be a promising airway device during CPR.

Several studies suggest that the Combitube is a useful airway device, especially in the pre-hospital setting [30-32]. Airway management using Combitube was significantly faster and the success rate higher compared to conventional endotracheal intubation. These results are comparable with those of Abo et. al [33]. The EasyTube performed comparable with the Combitube in our study. Therefore, we agree with Chenaitia et al., that the Easytube seems to be an effective alternate airway device [34].

As a limitation, our study was not designed or powered to show differences between particular supraglottic devices. Results of manikin-based studies are also limited in interpretation compared with humans, although we used an excellent manikin for airway management [35-37]. Detection of airway management associated problems like gastric inflation or aspiration is difficult with a manikin-based evaluation. However, evaluation of skill retention in identical airway conditions is only possible using a manikin. Another limitation of the study was, that participants were not trained in the management of advanced airways. Participants participated in a one-hour training covering endotracheal intubation and five supraglottic airway devices. This may be insufficient to expect any qualified handling of endotracheal intubation and may put endotracheal intubation in miscredit. However, results show that the learning curve for the supraglottic devices is much shorter. Furthermore, manikin studies cannot measure displacement of airway devices as a result of moving the patient. Generally, findings of manikin studies can be less reliable in real life and need to be confirmed in “real patients” [38].

In conclusion, endotracheal intubation in inexperienced hands is dangerous and could lead to fatal complications, like unrecognised misplacement. The success rate after a single training session is unacceptably low and required hands-off time during CPR far above the recommended maximum of 10 seconds.

In contrast, with a single training session and after a period without any training, the inexperienced EMT`s were able to perform successful airway management with acceptably low hands-off time by using one of the five supraglottic airway devices. If airway management must be performed by inexperienced personnel, EMT`s should not attempt endotracheal intubation and should consider supraglottic airway devices as the appropriate and valuable alternatives.

## Competing interest

Michael Frass invented the Combitube and has received royalties from Covidien. None of the other authors has a personal financial interest in this research.

## Authors’ contributions

CG contributed to the study design and acquisition of data, made substantial contributions to analysis and interpretation of data, drafted the manuscript, and revised it critically for important intellectual content. SN, PW, AR, HF contributed to aquisitation of data, and revised it critically for important intellectual content. DR and OK performed the statistical analysis and contributed to interpretation of data. MF made substantial contributions to analysis and interpretation of data and revised it critically for important intellectual content. KR contributed to the study design, made substantial contributions to analysis and interpretation of data, drafted the manuscript, and revised it critically for important intellectual content. All authors read and approved the final manuscript.
